# Late HIV diagnosis: trends, risk factors, and progress toward the 2025 target of <20% late diagnosis in 23 EU/EEA countries, 2022 to 2024

**DOI:** 10.2807/1560-7917.ES.2025.30.47.2500855

**Published:** 2025-11-27

**Authors:** Juliana Reyes-Urueña, Giorgia Stoppa, Federica Pizzolato, Gaetano Marrone, Disa Hansson, on behalf of the EU/EEA HIV network

**Affiliations:** 1 European Centre for Disease Prevention and Control (ECDC), Stockholm, Sweden; 2 Unit of Biostatistics, Epidemiology and Public Health, Department of Cardiac, Thoracic, Vascular Sciences and Public Health, University of Padova, Padua, Italy; 3 The members of the EU/EEA HIV network are listed in the Acknowledgements section at the end of the article.; * These authors contributed equally to this work.

**Keywords:** HIV, epidemiology, disease surveillance, HIV testing, late Diagnosis

## Abstract

In 2022–2024, 14,153 of 28,521 (49.6%) new HIV diagnoses in 23 European Union and Economic Area (EU/EEA) countries were late. In adjusted analyses, older age and migrant status increased late diagnosis risk. The proportion of late diagnoses was 2.6-fold higher among migrants with pre-migration HIV acquisition than post-migration. Late-diagnosed migrants with likely post-migration HIV acquisition were often women, ≥ 50-year-olds, heterosexuals, people who inject drugs, or from South and South-East Asia. The 2025 target of < 20% late diagnosis was unachieved.

The elimination of HIV as a public health threat is assessed with several indicators and defined targets [1]. One of these targets is that the proportion of new diagnoses made at a late stage should be < 20% by 2025 [1]. To characterise late diagnoses in the European Union and European Economic Area (EU/EEA) and assess the status toward the target there, as well as to identify risk factors for late diagnosis, we used 2022–2024 data on new HIV diagnoses in the EU/EEA. 

## People diagnosed with HIV at a late stage

Between 2022 and 2024, 75,156 HIV diagnoses were reported by 30 EU/EEA countries to the European Surveillance System (TESSy). Seven countries were excluded from the analysis as they could not differentiate new diagnoses from previously known positive diagnoses, or because most reported diagnoses were already known positive (n = 22,133). In the 23 remaining countries, we then excluded all previously known positive diagnoses (n = 11,400) and all cases with unknown status on whether the diagnosis was new or previously known (n = 4,318). This resulted in 37,305 diagnoses. We then also excluded diagnoses with unknown CD4^+^ T cell count or AIDS status (n = 8,426), and, due to small numbers, transgender cases and cases of unknown sex (n = 358), resulting in 28,521 cases included in our analyses. Children younger than 15 were excluded from the entire analysis. 

Late diagnosis is defined as a CD4^+^ T cell count < 350 cells/mm^3^ or an AIDS-defining event within 6 months of HIV diagnosis, unless there is evidence of recent infection [2]. For guiding targeted and equity-focused HIV testing, as well as policy and actions to meet the target and prevent HIV, we described people diagnosed with HIV according to sociodemographic and epidemiological characteristics, stratifying them by non-late or late diagnosis. This is presented in Table 1.

**Table 1:** Temporal, sociodemographic, epidemiological and geographical distribution of late and non-late HIV new diagnoses, as well as median CD4**^+^** T cell counts at diagnosis, in EU/EEA countries, 2022–2024 (n = 23 countries)^a^

Parameter	Women				Men				Total			
	Non late diagnosis		Late diagnosis^b^		Non late diagnosis		Late diagnosis^b^		Non late diagnosis		Late diagnosis^b^	
	n = 3,322		n = 3,947		n = 11,046		n = 10,206		n = 14,368		n = 14,153	
**Reporting year**												
2022	1,071	46.2%	1,246	53.8%	3,531	51.5%	3,323	48.5%	4,602	50.2%	4,569	49.8%
2023	1,167	44.3%	1,466	55.7%	3,785	52.2%	3,465	47.8%	4,952	50.1%	4,931	49.9%
2024	1,084	46.7%	1,235	53.3%	3,730	52.2%	3,418	47.8%	4,814	50.9%	4,653	49.1%
**Median age at HIV diagnosis, years (IQR)**												
Median (IQR)	35 (28, 45)		41 (33, 51)		35 (28, 45)		43 (33, 53)		35 (28, 45)		42 (33, 53)	
**Age category at HIV diagnosis in years**												
15–18	89	62.7%	53	37.3%	125	62.5%	75	37.5%	214	62.6%	128	37.4%
19–29	945	59.9%	633	40.1%	3,371	69.4%	1,485	30.6%	4,316	67.1%	2,118	32.9%
30–50	1,786	44.2%	2,259	55.8%	5,808	51.6%	5,453	48.4%	7,594	49.6%	7,712	50.4%
> 50	501	33.4%	1,001	66.6%	1,739	35.3%	3,192	64.7%	2,240	34.8%	4,193	65.2%
Unknown	1	50.0%	1	50.0%	3	75.0%	1	25.0%	4	66.7%	2	33.3%
**Mode of transmission**												
Sex between men	NA	NA	NA	NA	7,381	61.7%	4,574	38.3%	7,381	61.7%	4,574	38.3%
Heterosexual transmission (men)	NA	NA	NA	NA	2,353	39.7%	3,575	60.3%	2,353	39.7%	3,575	60.3%
Heterosexual transmission (women)	2,908	46.4%	3,359	53.6%	NA	NA	NA	NA	2,908	46.4%	3,359	53.6%
Injecting drug use	113	52.8%	101	47.2%	385	45.1%	469	54.9%	498	46.6%	570	53.4%
Mother to child transmission	23	74.2%	8	25.8%	10	40.0%	15	60.0%	33	58.9%	23	41.1%
Other routes^c^	12	44.4%	15	55.6%	5	29.4%	12	70.6%	17	38.6%	27	61.4%
Unknown	266	36.4%	464	63.6%	912	36.9%	1,561	63.1%	1,178	36.8%	2,025	63.2%
**Migration** ^d^												
Non migrants	944	49.9%	947	50.1%	6,371	52.9%	5,677	47.1%	7,315	52.5%	6,624	47.5%
Migrants born in the EU/EEA	98	40.5%	144	59.5%	663	56.3%	514	43.7%	761	53.6%	658	46.4%
Migrants born outside the EU/EEA	2,202	44.5%	2,743	55.5%	3,746	50.4%	3,681	49.6%	5,948	48.1%	6,424	51.9%
Unknown	78	40.8%	113	59.2%	266	44.3%	334	55.7%	344	43.5%	447	56.5%
**Region of origin**												
Reporting country	944	49.9%	947	50.1%	6,371	52.9%	5,677	47.1%	7,315	52.5%	6,624	47.5%
Western Europe	36	51.4%	34	48.6%	353	61.0%	226	39.0%	389	59.9%	260	40.1%
Central Europe	85	38.3%	137	61.7%	565	52.5%	512	47.5%	650	50.0%	649	50.0%
Eastern Europe	459	41.5%	647	58.5%	535	49.3%	550	50.7%	994	45.4%	1,197	54.6%
Sub-Saharan Africa	1,446	45.9%	1,702	54.1%	1,070	44.7%	1,323	55.3%	2,516	45.4%	3,025	54.6%
Latin America and Caribbean	161	46.5%	185	53.5%	1,082	56.3%	840	43.7%	1,243	54.8%	1,025	45.2%
South and South-east Asia	49	28.7%	122	71.3%	315	45.4%	379	54.6%	364	42.1%	501	57.9%
Other	64	51.6%	60	48.4%	489	57.3%	365	42.7%	553	56.5%	425	43.5%
Unknown	78	40.8%	113	59.2%	266	44.3%	334	55.7%	344	43.5%	447	56.5%
**Median CD4^+^ T cell count at diagnosis, cells/mm^3^ (IQR)**												
Median (IQR)	547 (430–706)		141 (47–243)		520 (412–678)		140 (45–250)		525 (416–684)		140 (46–249)	
**Reporting EU/EEA region**												
Eastern EU/EEA countries	131	40.1%	196	59.9%	413	48.5%	438	51.5%	544	46.2%	634	53.8%
Southern EU/EEA countries	974	40.0%	1,459	60.0%	3,626	43.6%	4,695	56.4%	4,600	42.8%	6,154	57.2%
Western EU/EEA countries	2,040	49.8%	2,053	50.2%	6,552	58.9%	4,572	41.1%	8,592	56.5%	6,625	43.5%
Northern EU/EEA countries	177	42.5%	239	57.5%	455	47.6%	501	52.4%	632	46.1%	740	53.9%

EEA: European Economic Area; EU: European Union; IQR: interquartile range; NA: not applicable; TESSy: the European Surveillance System.

^a^ Countries included in the analysis were Austria, Belgium, Croatia, Cyprus, Czechia, Denmark, Estonia, France, Germany, Greece, Iceland, Ireland, Italy, Latvia, Liechtenstein, Luxembourg, Malta, the Netherlands, Norway, Portugal, Slovakia, Slovenia, and Sweden. Of 30 EU/EEA reporting HIV diagnoses to TESSy, the following countries were excluded from the analysis: Bulgaria, Finland, Hungary, Lithuania, Poland, Romania, and Spain. These exclusions were due to the inability to classify diagnoses as new or previously known HIV positive, or because most reported diagnoses were already known positive.

^b^ For this analysis, late diagnosis was defined as having a CD4^+^ T cell count below 350 cells/mm^3^ within 6 months of HIV diagnosis or the presence of an AIDS-defining event regardless of CD4^+^ count; people with evidence of recent infection with CD4^+^ T cell count below 350 cells/mm^3^ were excluded from the numerator but retained in the denominator to calculate the proportion of late diagnosis.

^c^ Other routes of transmission included infection through blood transfusion and nosocomial transmission, referring to infections acquired in healthcare settings.

^d^ Migration was classified as non-migrant if the reporting country matched the country of birth or nationality. The migrant status was derived by using information on country of birth, nationality, and/or region of origin. When multiple variables were available, priority was given in the following order: country of birth, nationality, and then region of origin. Migrants were then classified into two categories based on their country or region of origin: those born in the EU/EEA and those born outside the EU/EEA. The non-migrant population was defined as people whose reported place of birth was the reporting country. We established two categories related to region. The first, ‘region of origin’ defines migrants’ birth region. The second category, ‘EU/EEA subregions’, defines sub-regions for the reporting countries within the EU/EEA as described in the country list in the Supplementary material.

Moreover, to assess the situation in the 23 EU/EEA countries regarding the target of <20% late diagnosis by 2025, we derived overall and annual proportions of late diagnoses between 2022 and 2024, relative, respectively, to the total diagnoses in this period and in each of its years (Table 1). Overall, 14,153 (14,153/28,521; 49.6%) HIV diagnoses were late. Furthermore, despite minor annual fluctuations, there was no reduction in either the number or the proportion of late diagnoses between 2022 and 2024 (Figure).

**Figure:**
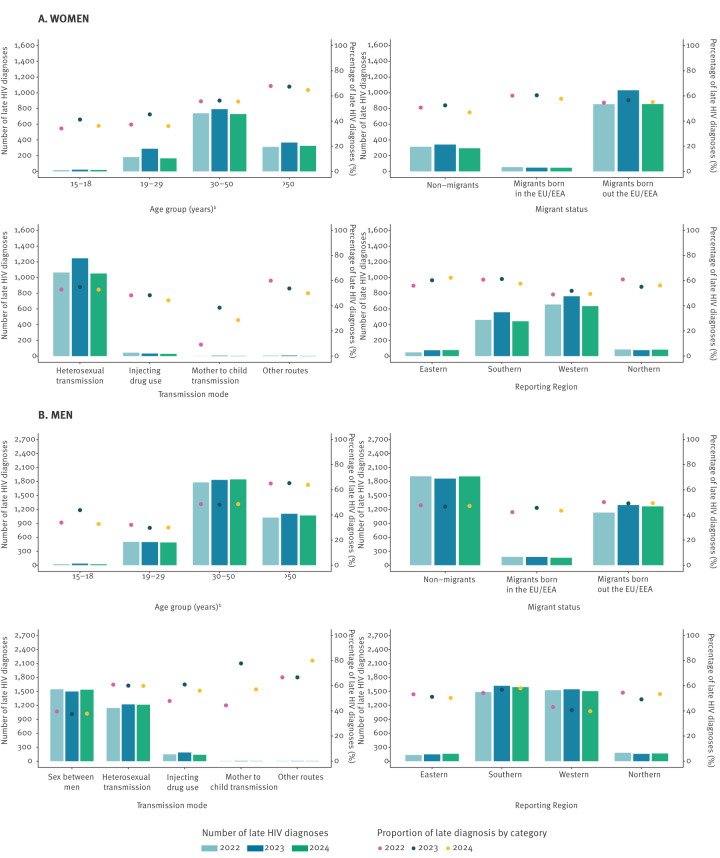
Annual numbers and proportions of late diagnoses among new HIV diagnoses, among (A) women and (B) men, stratified by sociodemographic characteristics in 23 EU/EEA countries^a^, 2022–2024 (n = 28,521 HIV diagnoses)

EEA: European Economic Area; EU: European Union; TESSy: the European Surveillance System.

^a^ Countries included in the analysis were Austria, Belgium, Croatia, Cyprus, Czechia, Denmark, Estonia, France, Germany, Greece, Iceland, Ireland, Italy, Latvia, Liechtenstein, Luxembourg, Malta, the Netherlands, Norway, Portugal, Slovakia, Slovenia, and Sweden. Of 30 EU/EEA reporting HIV diagnoses to TESSy, the following countries were excluded from the analysis: Bulgaria, Finland, Hungary, Lithuania, Poland, Romania, and Spain. These exclusions were due to the inability to classify cases as newly diagnosed or previously known positive, or because most reported diagnoses were already known positive.

^b^ For this analysis, we excluded six people reported with HIV diagnosis lacking age information.

## Risk factors for late HIV diagnosis

We then used a sex-stratified modified Poisson regression model to assess risk factors for late HIV diagnosis, including year of diagnosis, age group at HIV diagnosis, mode of transmission, migrant status, and reporting part of the EU/EEA as predictors (Table 2). Late HIV diagnosis showed similar patterns in women and men. The risk of late HIV diagnosis increased with age and was higher among people diagnosed in eastern EU/EEA countries compared with those in western EU/EEA countries. There was also a higher risk of late diagnosis among men in southern EU/EEA countries (prevalence ratio (PR): 1.17; 95% CI: 1.07–1.28) compared with men in eastern EU/EEA countries. Compared with non-migrants, being born outside the reporting country was also associated with an increased risk of late diagnosis, except for men born in another EU/EEA country. Men who have sex with men (MSM) had a lower risk of late diagnosis than men who acquired HIV through heterosexual transmission (PR: 0.74; 95% CI: 0.71–0.76).

**Table 2:** Sex-specific risk factors, as assessed by a modified Poisson model, for late HIV diagnosis in 23 EU/EEA countries, 2022–2024 (n = 24,783) ^a^

Parameters	Women (n = 6,418)		Men (n = 18,365)	
	PR	95% CI	PR	95% CI
**Year of diagnosis**				
2022	Ref.	Ref.	Ref.	Ref.
2023	1.04	0.98−1.10	0.98	0.95−1.02
2024	0.98	0.92−1.04	0.98	0.95−1.02
**Age group at HIV diagnosis in years**				
15–18	Ref.	Ref.	Ref.	Ref.
19–29	1.10	0.86−1.41	0.96	0.76−1.19
30–50	1.50	1.18−1.92	1.38	1.11−1.72
> 50	1.84	1.44−2.35	1.80	1.44−2.24
**Mode of transmission**				
Heterosexual transmission (men)	NA	NA	Ref.	Ref.
Heterosexual transmission (women)	Ref.	Ref.	NA	NA
Injecting drug use	0.93	0.80−1.08	0.95	0.89−1.02
Sex between men	NA	NA	0.74	0.71−0.76
Mother to child transmission	0.67	0.37−1.21	1.56	1.08−2.25
Other routes^b^	0.95	0.68−1.34	1.21	0.94−1.56
**Migration**				
Non-migrants	Ref.	Ref.	Ref.	Ref.
Migrants born in the EU/EEA	1.32	1.17−1.48	1.04	0.97−1.12
Migrants born outside the EU/EEA	1.27	1.20−1.34	1.15	1.11−1.19
**Reporting part of the EU/EEA **				
Eastern EU/EEA countries	Ref.	Ref.	Ref.	Ref.
Southern EU/EEA countries	0.99	0.88−1.11	1.17	1.07−1.28
Western EU/EEA countries	0.80	0.72−0.90	0.85	0.77−0.93
Northern EU/EEA countries	0.92	0.80−1.05	1.07	0.96−1.19

CI: confidence interval; EEA: European Economic Area; EU: European Union; PR: prevalence ratio; Ref.: reference; NA: not applicable.

^a^A total of 3,738 new HIV cases with unknown information on age, mode of transmission, and migrant status were excluded from the model. Countries included in the analysis were Austria, Belgium, Croatia, Cyprus, Czechia, Denmark, Estonia, France, Germany, Greece, Iceland, Ireland, Italy, Latvia, Liechtenstein, Luxembourg, Malta, the Netherlands, Norway, Portugal, Slovakia, Slovenia, and Sweden. Of 30 EU/EEA reporting HIV diagnoses to TESSy, the following countries were excluded from the analysis: Bulgaria, Finland, Hungary, Lithuania, Poland, Romania, and Spain. These exclusions were due to the inability to classify cases as newly diagnosed or previously known positive, or because most reported diagnoses were already known positive.

^b^ Other routes of transmission included infection through blood transfusion and nosocomial transmission, referring to infections acquired in healthcare settings.

## Late diagnosis among migrants, by whether HIV acquisition likely occurred pre- or post-migration

We classified HIV acquisition among migrants as pre- or post-migration using TESSy variables (country of birth, year of arrival, probable country/region of HIV acquisition, year of diagnosis, CD4^+^ T cell count at diagnosis, and markers of recent infection), prioritising the reported probable country/region of infection when available. When this was missing, we used markers of recent infection (see Table 3 footnote for a detailed definition), CD4^+^ T cell count, and time since arrival (≤ 1 year vs > 1 year) to infer likely timing of infection, and coded cases that could not be assigned as indeterminate. Among the 3,032 HIV diagnoses reported among migrants that were identified from 11 EU/EEA countries, 739 and 2,293 were classified as migrants who acquired HIV pre-migration and post-migration, respectively. Subsequently, we compared late and non-late diagnoses in each group. Further details are described in the footnote of Table 3.

**Table 3:** Late and non-late diagnoses among migrants reported with a new HIV diagnosis according to whether HIV was acquired pre- or post-migration in 11 countries in the EU/EEA, 2022–2024 (n = 3,032 diagnoses)

Parameters	Pre-migration HIV acquisition 739 (24.2%)				Post-migration HIV acquisition 2,293 (75.6%)			
	Non-late diagnosis		Late diagnosis		Non-late diagnosis		Late diagnosis	
	335 (45.3%)		404 (54.7%)		1,812 (79.0%)		481 (21.0%)	
**Year of diagnosis**								
2022	110	47.6%	121	52.4%	548	78.4%	151	21.6%
2023	113	43.3%	148	56.7%	630	80.5%	153	19.5%
2024	112	45.3%	135	54.7%	634	78.2%	177	21.8%
**Sex**								
Women	135	41.3%	192	58.7%	466	75.5%	151	24.5%
Men	200	48.5%	212	51.5%	1346	80.3%	330	19.8%
**Median age at HIV diagnosis, years (IQR)**								
Median (IQR)	33 (27–43)		39 (33–47)		33 (27–42)		39 (31–48)	
**Age group at HIV diagnosis in years**								
≤ 18	5	55.6%	4	44.4%	28	87.5%	4	12.5%
19–29	111	63.1%	65	36.9%	608	86.1%	98	13.9%
30–50	185	40.8%	268	59.2%	972	77.5%	283	22.5%
> 50	34	33.7%	67	66.3%	204	68.0%	96	32.0%
**Mode of transmission**								
Sex between men	125	62.2%	76	37.8%	938	83.3%	188	16.7%
Heterosexual transmission (men)	56	35.9%	100	64.1%	321	73.8%	114	26.2%
Heterosexual transmission (women)	125	42.2%	171	57.8%	425	75.0%	142	25.0%
Injecting drug use	5	29.4%	12	70.6%	16	69.6%	7	30.4%
Mother to child transmission	2	66.7%	1	33.3%	3	100.0%	0	0.0%
Other routes^a^	3	50.0%	3	50.0%	2	100.0%	0	0.0%
Unknown	19	31.7%	41	68.3%	107	78.1%	30	21.9%
**Migration**								
Migrants born in the EU/EEA	39	49.4%	40	50.6%	269	82.8%	56	17.2%
Migrants born outside the EU/EEA	296	44.8%	364	55.2%	1,543	78.4%	425	21.6%
**Region of origin**								
Western Europe	19	52.8%	17	47.2%	145	81.9%	32	18.1%
Central Europe	28	50.9%	27	49.1%	198	84.3%	37	15.7%
Eastern Europe	40	30.1%	93	69.9%	87	78.4%	24	21.6%
Sub-Saharan Africa	151	48.4%	161	51.6%	747	78.7%	202	21.3%
Latin America and Caribbean	64	56.1%	50	43.9%	282	79.2%	74	20.8%
South and South-east Asia	15	28.8%	37	71.2%	108	69.2%	48	30.8%
Other	18	48.6%	19	51.4%	245	79.3%	64	20.7%

IQR: interquartile range; EEA: European Economic Area; EU: European Union; PCI probable country of infection; TESSy: the European Surveillance System.

^a^ Other routes of transmission included infection through blood transfusion and nosocomial transmission, referring to infections acquired in healthcare settings.

For this analysis, we began with 37,305 newly reported HIV diagnoses from 23 countries. We restricted the sample to people with migrant status—both intra-EU/EEA migrants and migrants from non-EU/EEA countries (17,865). We excluded 277 cases with sex reported as transgender and cases with unknown sex. Next, we excluded countries in which > 50% of migrant cases had an unknown year of arrival, comprising 10,831 cases across 11 countries. The final sample comprised 7,034 cases from Austria, Belgium, Croatia, Denmark, Finland, France, Iceland, Ireland, Luxembourg, the Netherlands, Slovakia, and Slovenia.

Recent infection was defined as any of the following: primary HIV infection in the early phase after acquisition characterised by high HIV RNA levels; presence of p24 antigen before antibody development; symptoms of seroconversion illness; a documented negative HIV test within the previous 12 months; or evidence of recent infection based on Western blot results. This phase typically occurs 2 to 4 weeks after exposure and may present with low CD4^+^ T cell count [2]. To assess whether HIV acquisition among migrants occurred pre- or post-migration, we used variables available in TESSy: country of birth, year of arrival, PCI, year of diagnosis, CD4^+^ T cell count at diagnosis, and recent infection. Cases with PCI equal to the reporting country were classified as post-migration infections, whereas those with PCI outside the reporting country were considered pre-migration acquisition. When PCI was missing, classification was guided by recency evidence: infections were considered recent (≤ 12 months before diagnosis) if at least one marker was present in the recent infection variable People with recent infection and an estimated residence time of 1 year or less were classified as post-migration. For non-recent infections, cases with CD4^+^ T cell counts ≥ 350 cells/mm^3^ and residence longer than 1 year were considered post-migration, whereas those with residence of 1 year or less were classified as pre-migration. Cases that could not be classified based on these criteria were coded as indeterminate.

We calculated the number of residence years as the difference between the year of HIV diagnosis and the reported year of arrival in the reporting country on the 3,032 migrants who had this information available.

The median time from migration to diagnosis was 1 year (IQR: 0–3) for people classified as likely to have acquired HIV pre-migration, and 6 years (IQR: 2–16) for those with post-migration acquisition. The proportion of post-migration HIV acquisition appeared higher (75.6%) than pre-migration acquisition (24.4%). However, late diagnosis was more common after pre-migration HIV acquisition (54.7%) than after post-migration acquisition (21.0%). This pattern was sustained in all groups by year of diagnosis, age at diagnosis, mode of transmission, migration status, region of origin and reporting EU/EEA region (Table 3).

## Discussion

Late diagnosis increases the likelihood of onward community transmission [3]. Also, at the individual level, late diagnosis is associated with increased morbidity and mortality, greater loss of quality-adjusted life years and poorer outcomes for the individual [4] with substantially higher healthcare costs, largely due to hospitalisations and comedications [5].

In 2022–2024, 49.6% of new HIV diagnoses in the EU/EEA were diagnosed late. This is well above the regional targets of < 20% by 2025 and < 10% by 2030. The proportion remained stable between 2022 and 2024. The regional target of 20% should also be interpreted within a broader context: if a country (or region) achieves a 90% reduction in new HIV diagnoses through strong prevention efforts, the proportion of late diagnoses may increase and, paradoxically, this would reflect a positive development. Wider use of tests for recent HIV infection would help to clarify routine epidemiological data and better assess the impact of different policies.

Our results found that, older age and migration status were associated with increased likelihood of late diagnosis, while men who acquired HIV through sex between men were less likely to present late than men who acquired HIV through heterosexual contact. There was a lower risk of late diagnosis in western than eastern EU/EEA countries and, among men, a higher risk in southern EU/EEA countries. These results should guide identification of key populations for expanding and intensifying HIV testing efforts.

Among migrants, late diagnosis was more frequent in those who acquired HIV before migration (54.7%) than in those who did it after migration (21.0%), a crude 2.6-fold difference. However, another study reported a higher proportion (53.9%) of late diagnosis among migrants who acquired HIV post-migration [6], reinforcing concerns about late diagnosis in this population. Among migrants with late diagnosis who likely acquire HIV after migration, the highest proportions were observed among women, people aged ≥ 50 years, heterosexuals, people who inject drugs, and people originating from South and South-East Asia. These findings underscore the need for timely HIV testing among people who might acquire HIV post-migration to prevent delayed diagnosis and improve health outcomes.

Among migrants who acquired HIV before migration, late diagnosis is frequent (75.6%), with a median of 1 year from migration to diagnosis, suggesting a long period living with undiagnosed HIV before migration and limited access to testing in countries of origin or along migration routes, underlining the need to expand global access to HIV testing and antiretroviral therapy (ART). In the EU/EEA, late presentation on arrival is often not due to limited access to testing, but rather to structural barriers rooted in the socioeconomic and legal circumstances migrants face when seeking HIV testing [7]. To reduce onward transmission and improve health outcomes, it is essential to offer prevention services, routine HIV testing during health assessments for newcomers, migrant-inclusive community and entry-point voluntary testing, rapid linkage to care, and culturally and linguistically adapted services.

The high post-migration proportion points to ongoing transmission within host countries — through both heterosexual contact and sex between men — rather than being driven by importation alone. The post-migration HIV acquisition proportion calculated (75.6%) in this analysis exceeds single-country estimates from Norway (14%) [8] and Sweden (20%) [9] and prior TESSy estimates (67% to 72%) from 2009 [10]. These differences likely reflect variations in the timing of estimates, the countries included in the analysis, migration patterns, population composition, testing policies, and methodological approaches (e.g. linked registry data vs model-based estimates).

Barriers to testing include factors identified in other studies, such as low perceived risk, limited HIV-related knowledge, and stigma among heterosexuals and older adults [11]. Additionally, age-related stereotypes — such as the belief that older people are not sexually active or at risk — further reduce the likelihood of being offered testing or included in outreach efforts [12,13]. In primary care, risk is often underestimated, and testing is frequently initiated mainly upon patient request [14]. Among migrants, overlapping individual (low perceived risk, limited knowledge, stigma), sociocultural (language barriers, cultural norms, gendered assumptions), and structural factors (legal status, insurance, bureaucracy, precarious living) contribute to both post-migration transmission and late diagnosis [7]. It is also important to note that HIV testing is often missed during healthcare visits, even when patients present with indicator conditions that should trigger testing according to guidelines [15].

To reduce late HIV diagnosis, efforts should focus on expanding community-based, outreach and mobile services, as well as making self-testing widely available and affordable [16]. Routinising and normalising HIV testing for example through routine opt-out offers in primary care, emergency departments, antenatal care and prisons. In addition, indicator-condition–guided testing can help to detect infections missed in the recent phase. Structural barriers can be reduced by eliminating user fees, simplifying eligibility testing criteria, safeguarding confidentiality, and addressing stigma through provider training and public awareness campaigns. Improving HIV outcomes also requires integrated testing with rapid linkage to care (including same-day initiation of ART). HIV testing strategies should be tailored to each country’s epidemiological context to ensure that testing efforts effectively reach the populations most at risk, while also considering social determinants that may undermine overall effectiveness.

This study has several limitations. First, late diagnosis may be overestimated if recent infections were misclassified or underestimated if previously known HIV infections were erroneously recorded as new [17]. Second, the findings are not fully generalisable as not all EU/EEA countries contributed data, particularly for estimates of pre-and post- migration HIV acquisition. Third, the modified Poisson model did not account for pre-post migration HIV acquisition, so the higher risk seen among migrants may partially reflect migrants with pre-migration acquisition who were diagnosed late. Fourth, the estimation of pre- post-migration HIV acquisition was based on a small number of cases from 11 countries and therefore not representative of the entire EU/EEA. Finally, use of the probable country of infection variable could overestimate pre-migration acquisition as it has been found to be biased towards clinicians’ assumptions related to the patients’ country of origin rather than on biological or in-depth patient histories. Although previous work shows similar post-migration proportions when using clinical records or CD4^+^-based models [18], estimates should be interpreted with caution.

## Conclusion

Nearly half of new HIV diagnoses in 23 EU/EEA countries between 2022 and 2024 were made late, far exceeding regional targets and highlighting inequalities in accessing testing by age, transmission mode, migration status, and reporting EU/EEA region. Strengthening and normalising HIV testing — particularly through inclusive, accessible, and stigma-free approaches — is essential to reduce late diagnosis and achieve 2030 target.

## Data Availability

Study materials and raw data are available upon request.

## Supplementary Data

129000 SupplementaryMaterial
