# Supplementary material for: Late HIV diagnosis: trends, risk factors, and progress toward the 2025 target of <20% late diagnosis in 23 EU/EEA countries, 2022 to 2024
Source: Euro Surveill. 2025 Nov 27;30(47):2500855. doi: 10.2807/1560-7917.ES.2025.30.47.2500855 (PMC12788719; doi:10.2807/1560-7917.ES.2025.30.47.2500855)
Supplement: SupplementaryMaterial [file 25-00855_Supplementary_material.pdf]

This supplementary material is hosted by *Eurosurveillance* as supporting information alongside the article [Late HIV diagnosis: trends, risk factors, and progress toward the 2025 target of <20% late diagnosis in 23 EU/EEA countries, 2022 to 2024], on behalf of the authors, who remain responsible for the accuracy and appropriateness of the content. The same standards for ethics, copyright, attributions and permissions as for the article apply. Supplements are not edited by *Eurosurveillance* and the journal is not responsible for the maintenance of any links or email addresses provided therein.

Table S1. Country distribution by reporting subregion

Reporting subregions			
**Eastern EU/EEA subregion**	**Southern EU/EEA subregion**	**Western EU/EEA subregion**	**Northern EU/EEA subregion**
*Bulgaria (BG)<br>Czech Republic (CZ)<br>*Hungary (HU)<br>*Poland (PL)<br>*Romania (RO)<br>Slovakia (SK)<br>Estonia (EE)<br>Latvia (LV)<br>*Lithuania (LT)	*Croatia (HR)<br>Cyprus (CY)<br>Greece (EL)<br>*Italy (IT)<br>*Malta (MT)<br>Portugal (PT)<br>*Slovenia (SI)<br>*Spain (ES)	Austria (AT)<br>Belgium (BE)<br>France (FR)<br>Germany (DE)<br>Liechtenstein (LI)<br>Luxembourg (LU)<br>Netherlands (NL)	Denmark (DK)<br>*Finland (FI)<br>Iceland (IS)<br>Ireland (IE)<br>Norway (NO)<br>Sweden (SE)

*These countries were excluded from the analyses because they were unable to classify diagnoses as either new or previously positive.

Table S2. Country distribution by region of origin based on UNAIDS designation

Geographical region of origin			
**Western Europe**	**Central Europe**	**Eastern Europe**	**Latin America and Caribbean**
Andorra (AD)<br>Austria (AT)<br>Belgium (BE)<br>Denmark (DK)<br>Finland (FI)<br>Faroe Islands (FO)<br>France (FR)<br>Germany (DE)<br>Greenland (GL)<br>Greece (EL)<br>Iceland (IS)<br>Ireland (IE)<br>Israel (IL)<br>Italy (IT)<br>Liechtenstein (LI)<br>Luxembourg (LU)<br>Malta (MT)<br>Monaco (MC)<br>Netherlands (NL)<br>Norway (NO)<br>Portugal (PT)<br>San Marino (SM)<br>Spain (ES)<br>Sweden (SE)<br>Switzerland (CH)<br>United Kingdom (UK)	Bosnia and Herzegovina (BA)<br>Bulgaria (BG)<br>Croatia (HR)<br>Cyprus (CY)<br>Czech Republic (CZ)<br>Hungary (HU)<br>North Macedonia (MK)<br>Poland (PL)<br>Romania (RO)<br>Serbia and Montenegro (CS) *(no longer in use)*<br>Slovakia (SK)<br>Slovenia (SI)<br>Turkey (TR)<br>Yugoslavia (YU) *(no longer in use)*<br>Kosovo (XK)<br>Serbia (RS)	Armenia (AM)<br>Azerbaijan (AZ)<br>Belarus (BY)<br>Estonia (EE)<br>Georgia (GE)<br>Kazakhstan (KZ)<br>Kyrgyzstan (KG)<br>Latvia (LV)<br>Lithuania (LT)<br>Montenegro (ME)<br>Moldova (MD)<br>Russia (RU)<br>Tajikistan (TJ)<br>Turkmenistan (TM)<br>Ukraine (UA)<br>Uzbekistan (UZ)	Antigua and Barbuda (AG)<br>Netherlands Antilles (AN)<br>Argentina (AR)<br>Uruguay (UY)<br>Bahamas (BS)<br>Barbados (BB)<br>Cuba (CU)<br>Dominica (DM)<br>Dominican Republic (DO)<br>Grenada (GD)<br>Guadeloupe (GP)<br>Haiti (HT)<br>Jamaica (JM)<br>Saint Kitts and Nevis (KN)<br>Saint Lucia (LC)<br>Montserrat (MS)<br>Martinique (MQ)<br>Trinidad and Tobago (TT)<br>Saint Vincent and the Grenadines (VC)<br>Belize (BZ)<br>Bolivia (BO)<br>Brazil (BR)<br>Chile (CL)<br>Colombia (CO)<br>Costa Rica (CR)<br>Ecuador (EC)<br>El Salvador (SV)<br>French Guiana (GF)<br>Guatemala (GT)<br>Guyana (GY)<br>Honduras (HN)<br>Mexico (MX)<br>Nicaragua (NI)<br>Panama (PA)<br>Paraguay (PY)<br>Peru (PE)<br>Puerto Rico (PR)<br>Turks and Caicos (TC)

			Venezuela (VE)<br>Suriname (SR)

**Geographical region of origin**			
**Sub Saharan Africa**		**South and Southeast Asia**	**Other**
Angola (AO)<br>Benin (BJ)<br>Botswana (BW)<br>Burkina Faso (BF)<br>Burundi (BI)<br>Cameroon (CM)<br>Central African Republic (CF)<br>Cape Verde (CV)<br>Comoros (KM)<br>Republic of the Congo (CG)<br>Democratic Republic of the Congo (CD)<br>Ivory Coast (CI)<br>Djibouti (DJ)<br>Equatorial Guinea (GQ)<br>Eritrea (ER)<br>Ethiopia (ET)<br>Gabon (GA)<br>Gambia (GM)<br>Ghana (GH)<br>Guinea (GN)<br>GuineaBissau (GW)<br>Kenya (KE)	Lesotho (LS)<br>Liberia (LR)<br>Madagascar (MG)<br>Malawi (MW)<br>Mali (ML)<br>Mauritania (MR)<br>Mauritius (MU)<br>Mozambique (MZ)<br>Namibia (NA)<br>Namibia (NAM)<br>*(alternative code for Namibia)*<br>Nigeria (NG)<br>Rwanda (RW)<br>Senegal (SN)<br>Sierra Leone (SL)<br>Somalia (SO)<br>South Africa (ZA)<br>Eswatini (SZ)<br>Tanzania (TZ)<br>Togo (TG)<br>Uganda (UG)<br>Zambia (ZM)<br>Zimbabwe (ZW)<br>Mayotte (YT)<br>Seychelles (SC)<br>Chad (TD)<br>Niger (NE)<br>South Sudan (SS)	China (CN)<br>Macau (MO)<br>Fiji (FJ)<br>Japan (JP)<br>North Korea (KP)<br>South Korea (KR)<br>Mongolia (MN)<br>Papua New Guinea (PG)<br>Solomon Islands (SB)<br>Tonga (TO)<br>Taiwan (TW)<br>Bangladesh (BD)<br>Bhutan (BT)<br>Brunei (BN)<br>Cambodia (KH)<br>Hong Kong (HK)<br>India (IN)<br>Indonesia (ID)<br>Iran (IR)<br>Laos (LA)<br>Malaysia (MY)<br>Maldives (MV)<br>Myanmar (MM)<br>Nepal (NP)<br>Pakistan (PK)<br>Philippines (PH)<br>Singapore (SG)<br>Sri Lanka (LK)<br>Thailand (TH)<br>Vietnam (VN)<br>Timor Leste (TL)<br>Afghanistan (AF)	Algeria (DZ)<br>Bahrain (BH)<br>Egypt (EG)<br>Iraq (IQ)<br>Jordan (JO)<br>Kuwait (KW)<br>Lebanon (LB)<br>Libya (LY)<br>Morocco (MA)<br>Oman (OM)<br>Qatar (QA)<br>Saudi Arabia (SA)<br>Sudan (SD)<br>Soviet Union (SU)<br>*(no longer in use)*<br>Syria (SY)<br>Tunisia (TN)<br>United Arab Emirates (AE)<br>Yemen (YE)<br>United States (US)<br>Christmas Island (CX)<br>New Zealand (NZ)<br>Canada (CA)<br>Australia (AU)<br>Palestine (PS)
